# A real-world clinicopathological model for predicting pathological complete response to neoadjuvant chemotherapy in breast cancer

**DOI:** 10.3389/fonc.2024.1323226

**Published:** 2024-02-14

**Authors:** Shan Fang, Wenjie Xia, Haibo Zhang, Chao Ni, Jun Wu, Qiuping Mo, Mengjie Jiang, Dandan Guan, Hongjun Yuan, Wuzhen Chen

**Affiliations:** ^1^ Center for Rehabilitation Medicine, Rehabilitation & Sports Medicine Research Institute of Zhejiang Province, Department of Rehabilitation Medicine, Zhejiang Provincial People’s Hospital (Affiliated People’s Hospital), Hangzhou Medical College, Hangzhou, Zhejiang, China; ^2^ General Surgery, Cancer Center, Department of Breast Surgery, Zhejiang Provincial People’s Hospital (Affiliated People’s Hospital), Hangzhou Medical College, Hangzhou, Zhejiang, China; ^3^ Cancer Center, Department of Radiation Oncology, Zhejiang Provincial People’s Hospital (Affiliated People’s Hospital), Hangzhou Medical College, Hangzhou, Zhejiang, China; ^4^ Department of Breast Surgery (Surgical Oncology), Second Affiliated Hospital, Zhejiang University School of Medicine, Hangzhou, China; ^5^ Department of Radiotherapy, The First Affiliated Hospital of Zhejiang Chinese Medical University (Zhejiang Provincial Hospital of Chinese Medicine), Hangzhou, China; ^6^ Department of Oncology, Lanxi People’s Hospital, Jinhua, China

**Keywords:** breast cancer, predictive model, neoadjuvant chemotherapy, pathological complete response, prognosis

## Abstract

**Purpose:**

This study aimed to develop and validate a clinicopathological model to predict pathological complete response (pCR) to neoadjuvant chemotherapy (NAC) in breast cancer patients and identify key prognostic factors.

**Methods:**

This retrospective study analyzed data from 279 breast cancer patients who received NAC at Zhejiang Provincial People’s Hospital from 2011 to 2021. Additionally, an external validation dataset, comprising 50 patients from Lanxi People’s Hospital and Second Affiliated Hospital, Zhejiang University School of Medicine from 2022 to 2023 was utilized for model verification. A multivariate logistic regression model was established incorporating clinical, ultrasound features, circulating tumor cells (CTCs), and pathology variables at baseline and post-NAC. Model performance for predicting pCR was evaluated. Prognostic factors were identified using survival analysis.

**Results:**

In the 279 patients enrolled, a pathologic complete response (pCR) rate of 27.96% (78 out of 279) was achieved. The predictive model incorporated independent predictors such as stromal tumor-infiltrating lymphocyte (sTIL) levels, Ki-67 expression, molecular subtype, and ultrasound echo features. The model demonstrated strong predictive accuracy for pCR (C-statistics/AUC 0.874), especially in human epidermal growth factor receptor 2 (HER2)-enriched (C-statistics/AUC 0.878) and triple-negative (C-statistics/AUC 0.870) subtypes, and the model performed well in external validation data set (C-statistics/AUC 0.836). Incorporating circulating tumor cell (CTC) changes post-NAC and tumor size changes further improved predictive performance (C-statistics/AUC 0.945) in the CTC detection subgroup. Key prognostic factors included tumor size >5cm, lymph node metastasis, sTIL levels, estrogen receptor (ER) status and pCR. Despite varied pCR rates, overall prognosis after standard systemic therapy was consistent across molecular subtypes.

**Conclusion:**

The developed predictive model showcases robust performance in forecasting pCR in NAC-treated breast cancer patients, marking a step toward more personalized therapeutic strategies in breast cancer.

## Introduction

Breast cancer is a primary cause of cancer-related mortality among women globally (1, 2). Despite strides in therapeutic approaches, the death rate associated with advanced stages of the disease remains distressingly high (3–5). In this context, neoadjuvant chemotherapy (NAC) has risen to prominence as a crucial measure for gauging therapeutic effectiveness. It has shown notable efficacy in enhancing event-free survival (EFS) and overall survival (OS) rates, particularly among patients who achieve pathological complete response (pCR) during treatment (6–8). Additionally, the customization of post-neoadjuvant treatments has been promising in augmenting long-term outcomes for patients who do not attain pCR (9, 10). This underscores the importance of accurately predicting pCR in breast cancer patients, a critical factor in optimizing treatment strategies and improving survival prospects.

Extensive research has focused on identifying the determinants of pathologic complete response (pCR) and prognosis following NAC in breast cancer. Tumor-infiltrating lymphocytes (TILs), recognized as key regulators within the tumor microenvironment (11–14). The TILs could be classified into two types: intratumoral (iTILs) and stromal (sTILs), depending on whether they are located within the tumor nest or embedded in the tumor stroma (15, 16). Studies have indicated that sTILs are a better and more reproducible biomarker than iTILs (15, 17). TIL levels, particularly sTILs, have been highlighted as predictors of response to neoadjuvant chemotherapy in breast cancer (18, 19).

Circulating tumor cells (CTCs), which are a subset of tumor cells shed into the peripheral blood as a result of tumor tissue instability, have been identified as independent predictors of pCR and prognosis (20, 21). Additionally, traditional diagnostics, such as ultrasound paired with molecular markers like CA15-3 and CEA, also correlate with pCR and prognosis (22–24). Recent advances highlight the significance of combining imaging, pathology, and hematology in assessing treatment efficacy, as shown in Wang et al.’s model which integrates clinical and radiomics features such as background parenchymal enhancement (BPE), human epidermal growth factor receptor-2 (HER-2) status, and the Ki-67 index (25).

Despite the considerable accuracy of existing models, their limitations in specificity underscore the ongoing critical need for research focused on determining pCR status following NAC in breast cancer. Our study is directed towards developing an integrated predictive model that combines clinical (ultrasound), pathological (Ki-67 expression, molecular subtype), and novel biomarkers (sTIL levels, CTCs). We expect this comprehensive model to outperform individual indicators in predicting pCR with greater accuracy, thus providing a foundation for personalized breast cancer treatment strategies.

## Materials and methods

### Study participants

**Figure 1:**
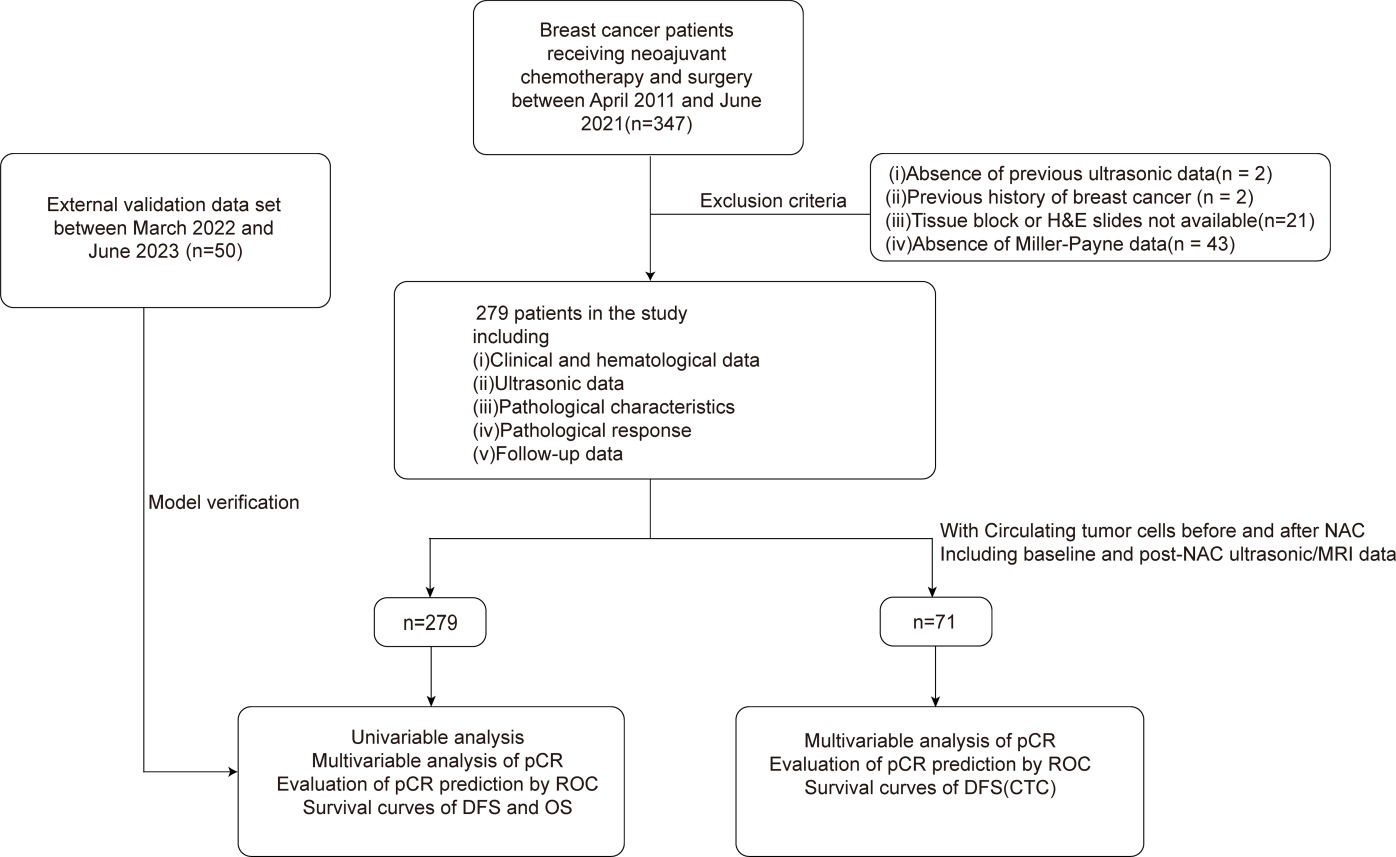
Study profile of 347 patients who received NAC and subsequently underwent surgery between April 2011 and June 2021. 279 patients met the eligibility criteria and were enrolled in this study. Additionally, a validation set comprising 50 patients was included. Subgroup analysis was performed in 71 patients with CTC data. NAC, Neoadjuvant chemotherapy; CTC, circulating tumor cell.

Between April 2011 and June 2021, our study, adhering to rigorous clinical, ultrasonic, and pathological criteria, enrolled 279 female patients from the breast cancer database of Zhejiang Provincial People’s Hospital (ZJPPH-BCDB). These patients, all pathologically confirmed with breast cancer, had undergone neoadjuvant chemotherapy (NAC) prior to surgery. This cohort was refined from an initial pool of 347 patients, as depicted in **Figure 1**. To validate our model, we further gathered an external dataset comprising 50 patients from Lanxi People’s Hospital (Jinhua, China) and the Second Affiliated Hospital, Zhejiang University School of Medicine (Hangzhou, China) between March 2022 and June 2023. We ensured that all participants had no prior malignant disease or contraindications to chemotherapy. A subset of 71 participants was assessed for CTCs both pre- and post-NAC. This study received approval from the ethics committees of all the institutions involved, with informed consent obtained from participants with CTC detection, and adherence to all relevant guidelines and regulations was strictly maintained throughout the research process.

### Data collection

Clinical, hematological, and ultrasonic data were systematically collected. Clinical parameters included age, body mass index (BMI), menopausal status, red cell distribution width (RDW), platelet distribution width (PDW), mean platelet volume (MPV), carcinoembryonic antigen (CEA), cancer-associated antigen (CA) 125, and CA153. Metrics derived from ultrasound (US) encompass tumor dimensions, geometric form, internal echogenicity, and the ratio of width to height. Pathological data, sourced from pre-NAC core-needle biopsy specimens, encompassed Ki-67, estrogen receptor (ER), progesterone receptor (PR), and human epidermal growth factor receptor 2 (HER2) expression. These were assessed using the American Society of Clinical Oncology and the American College of Pathologist guidelines, employing immunohistochemical methods (26, 27). sTIL levels, classified as low, intermediate, or high (0-10%, 11-39%, and ≥40%, respectively), were determined based on recommendations from previous research (16, 17). For the CTC detection subgroup, CTC data was collected pre- and post-NAC in 71 patients. Metrics from magnetic resonance imaging (MRI) capture tumor dimensions, structural configuration, signal intensity variations, and patterns of contrast enhancement in the CTC detection subgroup. Patients with ≥1 CTC per 7.5 mL of blood were categorized as CTC-positive (28). Pathological response was assessed using the Miller-Payne system, with a grade of 5 indicating pCR (29). Follow-up was conducted through phone checks and outpatient visits, with overall survival (OS) and disease-free survival (DFS) being defined according to standard clinical endpoints (30).

### Statistical analysis

Analyses were conducted using SPSS (IBM SPSS 26.0, SPSS Inc.). Categorical data, represented as percentages, were assessed using appropriate chi-square tests. Continuous data were presented either as means ± SD or as medians with interquartile ranges (P50 [P25, P75]), depending on their distribution, and analyzed using the t-test or Mann-Whitney U test, respectively. Significant variables from univariate analyses (p < 0.05) were advanced to multivariate logistic regression. Stepwise logistic regression was employed, with the Hosmer-Lemeshow test evaluating model fit. The ROC and AUC were used to appraise model calibration and discrimination. Survival analyses were conducted using Kaplan-Meier curves, with significance ascertained via the log rank (Mantel–Cox) test. A threshold of p < 0.05 was set for statistical significance.

## Results

### Clinical features and pCR achievement

Of the 279 breast cancer patients, 27.96% (78/279) achieved a pCR after NAC, with the clinical, hematological, ultrasonic, and pathological data (**Table 1**). No significant differences were noted between pCR and non-pCR patients in terms of age, BMI, and menopausal status (p > 0.05). However, CEA levels significantly varied between the groups (p = 0.011), while no significant associations were observed for RDW, PDW, MPV, CA125, or CA153 (p > 0.05). In the context of ultrasonic findings, tumor size and tumor posterior echo were significantly associated with pCR status (p = 0.017 and p = 0.013, respectively).

**Table 1:** Patient characteristics of pCR and non-pCR.

Clinicopathological Characteristics		Non-pCR (n=201)	pCR (n=78)	T/Z/χ^2^	p
MP Grade	1	6	–		
	2	62	–		
	3	85	–		
	4	48	–		
	5	–	78		
Age (years)		50.02 ± 10.26	48.26 ± 10.02	1.3	0.195
BMI		23.68 ± 3.51	24.41 ± 5.06	-1.377	0.17
BI (US,n=105/39)		0.75 ± 0.11	0.76 ± 0.11	-0.476	0.635
RDW		13.11 ± 1.34	13.26 ± 2.11	-0.695	0.488
PDW(fl)		13.66 ± 2.84	13.75 ± 2.75	-0.228	0.820
MPV(fl)		11.00 ± 1.23	11.03 ± 1.22	-0.169	0.866
CEA		2.2 (1.4,3.5)	1.8 (1.1,2.3)	2.541	0.011
CA125		13.8 (9.6,20.5)	14.95 (11,21.5)	-0.891	0.373
CA153		18.1 (12.9,26.9)	16.7 (11.9,24.4)	1.025	0.305
Ki-67 expression(%)		25 (15,40)	33 (25,60)	-3.362	0.001
sTILs (%)		15 (5,20)	30 (20,40)	-8.562	<0.001
Menopausal status	Premenopausal	104 (51.7%)	48 (61.5%)	2.175	0.140
	Postmenopausal	97 (48.3%)	30 (38.5%)		
Tumor size (US)	≤5cm	155 (77.1%)	70 (89.7%)	5.742	0.017
	>5cm	46 (22.9%)	8 (10.3%)		
Tumor shape (US)	Regular	7 (3.5%)	2 (2.6%)	0.000	0.990^a^
	Irregular	194 (96.5%)	76 (97.4%)		
Tumor internal echo (US)	Uniform	17 (8.5%)	4 (5.1%)	0.481	0.488^a^
	Uneven	184 (91.5%)	74 (94.9%)		
Tumor aspect ratio (US)	≤1	191 (95.0%)	69 (88.5%)	3.814	0.051
	>1	10 (5.0%)	9 (11.5%)		
Calcification (US)	Negative	85 (42.3%)	33 (42.3%)	0.000	0.998
	Positive	116 (57.7%)	45 (57.7%)		
Tumor posterior echo (US)	Attenuation	63 (31.3%)	12 (15.4%)	7.836	0.013^b^
	Unchanged	136 (67.7%)	65 (83.3%)		
	Enhancement	2 (1.0%)	1 (1.3%)		
Abnormal blood flow signal (US)	Negative	31 (15.4%)	14 (17.9%)	0.265	0.607
	Positive	170 (84.6%)	64 (82.1%)		
Lymphatic metastasis (US)	Negative	82 (40.8%)	25 (32.1%)	1.818	0.178
	Positive	119 (59.2%)	53 (67.9%)		
ER status	Negative	75 (37.3%)	47 (60.3%)	16.303	<0.001
	Weakly positive	24 (11.9%)	12 (15.4%)		
	Strongly positive	102 (50.7%)	19 (24.4%)		
PR status	Negative	81(40.3%)	48 (61.5%)	10.970	0.004
	Weakly positive	64 (31.8%)	19 (24.4%)		
	Strongly positive	56 (27.9%)	11 (14.1%)		
HER2 status	Negative	42 (20.9%)	17 (21.8%)	24.881	<0.001
	Weakly positive	91 (45.3%)	12 (15.4%)		
	Strongly positive	68 (33.8%)	49 (62.8%)		
Four molecular subtypes	ER- PR- HER2-	39 (19.4%)	21 (26.9%)	33.859	<0.001
	ER/PR+ HER2-	94 (46.8%)	8 (10.3%)		
	ER/PR- HER2+	26 (12.9%)	22 (28.2%)		
	ER/PR+ HER2+	42 (20.9%)	27 (34.6%)		
Ki-67 expression(%)	<14%	47 (23.7%)	8 (10.3%)	6.373	0.012
	≥14%	151 (76.3%)	70 (89.7%)		
Chemotherapy	Non-E	30 (14.9%)	24 (30.8%)	9.037	0.003
	E	171 (85.1%)	54 (69.2%)		
sTILs	Low	92 (45.8%)	5 (6.4%)	67.553	<0.001
	Intermediate	100 (49.8%)	45 (57.7%)		
	High	9 (4.5%)	28 (35.9%)		

MP: Miller-Payne grade, BI: Blood flow resistance index of US, US: ultrasound, RDW: red blood cells distribution width, PDW: platelet distribution width, MPV: mean platelet volume, sTILs: stromal tumor infiltrating lymphocytes, Low (sTILs ≤ 10%), Intermediate (10<sTILs<40%), High (sTILs≥40%); ER: estrogen receptor, PR: progesterone receptor, HER2: human epidermal growth factor receptor-2; Data format: x ± s, P50 (P25, P75), (n,%); a: Continuity Correction of Pearson Chi-Square; b: Fisher’s Exact Test.

Patients who achieved pCR predominantly presented with ER-negative (p < 0.001), PR-negative (p = 0.004), and HER2-positive (p < 0.001) phenotypes. Disparities in pCR rates were evident among molecular subtypes (p < 0.001), with the highest pCR rate in the HER2-enriched (ER- PR- HER2+) subgroup (45.83%, 22/48) and the lowest in the ER/PR+ HER2- subgroup (7.8%, 8/102) (**Table 2**). There were also significant differences in Ki-67 expression and sTIL levels between patients who realized pCR and those who did not (Ki-67: p < 0.05, sTIL levels: p < 0.001).

**Table 2:** Molecular subtypes and pCR rate.

Molecular subtypes	n	non-pCR	pCR	χ^2^	p
ER- PR- HER2-	60	39 (65.0%)	21 (35.0%)	33.859	<0.001
ER/PR+ HER2-	102	94 (92.2%)	8 (7.8%)		
ER/PR- HER2+	48	26 (54.2%)	22 (45.8%)		
ER/PR+ HER2+	69	42 (60.9%)	27 (39.1%)		

ER, estrogen receptor; PR, progesterone receptor; HER2, human epidermal growth factor receptor-2.

### Overall predictive model of pCR

Multivariate analysis utilizing the significant factors from **Table 1** identified sTIL levels, Ki-67 expression, tumor posterior echo, and molecular subtypes as independent predictors of pCR (all p < 0.05) (**Table 3**). The predictive logistic model based on these factors achieved a good fit (Hosmer–Lemeshow test, p = 0.962).

**Table 3:** Multivariate regression analysis for pCR (n=279).

	B	Wald	OR	95%CI	p
Molecular subtypes					
ER/PR+ HER2+		16.614	REF		0.001
ER- PR- HER2-	-2.185	13.799	0.112	0.035-0.356	<0.001
ER/PR+ HER2-	-1.144	5.506	0.319	0.123-0.828	0.019
ER/PR- HER2+	-0.99	4.082	0.372	0.142-0.971	0.043
sTILs	0.145	40.29	1.156	1.105-1.209	<0.001
Ki-67 expression	0.019	5.372	1.019	1.003-1.036	0.02
Posterior echo of US	0.91	5.335	2.484	1.148-5.374	0.021
Constant	-3.668	35.04	0.026		<0.001

sTILs, stromal tumor infiltrating lymphocytes; US, ultrasound; Logistic model, Hosmer–Lemeshow test validity, P = 0.962.

**Figure 2:**
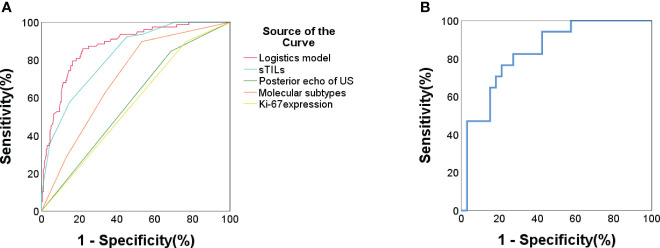
**(A)** ROC curve for pCR prediction for all included patients (n = 279). Logistic prediction model (AUC/C-statistics = 0.874) and the 4 components factors: sTIL levels (AUC/C-statistics =0.822); Ki67 expression (AUC/C-statistics = 0.629); Molecular subtypes (AUC/C-statistics = 0.705); Tumor posterior echo (AUC/C-statistics = 0.58). **(B)** ROC curve for pCR prediction of external validation data set in Lanxi People’s Hospital and Second Affiliated Hospital (n = 50). Logistic prediction model (AUC/C-statistics = 0.836).

ROC analysis demonstrated a C-statistics/AUC of 0.874 (95% CI: 0.829-0.918) for the multivariate model. Individual predictors’ performances were: sTIL levels C-statistics/AUC = 0.822, molecular subtypes C-statistics/AUC = 0.705, Ki-67 expression C-statistics/AUC = 0.629, and tumor posterior echo C-statistics/AUC = 0.580 (**Table 4**, **Figure 2A**). Notably, the model’s predictive accuracy was highest for the HER2-enriched and triple negative breast cancer (TNBC, ER- PR- HER2-) subtypes (C-statistics/AUC of 0.878 and 0.870 respectively) (**Supplementary Figure S1**). To validate this predictive model, an external validation set comprising 50 patients was utilized. The comparison between the primary set and the external validation dataset revealed no significant differences in terms of sTIL levels, Ki-67 expression, tumor posterior echo, and molecular subtypes (p > 0.05) (**Table 5**). The model exhibited robust performance in this external validation cohort (C-statistics/AUC =0.836, 95% CI: 0.724-0.948) (**Figure 2B**).

**Table 4:** ROC analysis for pCR(n=279).

Characteristics	C-statistics/AUC	95%CI	Sensitivity	Specificity	p
Ki67 expression	0.629	0.557-0.701	0.718	0.507	0.001
sTILs	0.822	0.772-0.873	0.923	0.547	<0.001
Posterior echo of US	0.58	0.508-0.652	0.846	0.313	0.038
Molecular subtypes	0.705	0.641-0.769	0.897	0.468	<0.001
Logistic model	0.874	0.829-0.918	0.859	0.781	<0.001

ROC analysis of the Logistic model and 4 consisting factors for prediction of pCR. US: ultrasound; sTILs: stromal tumor infiltrating lymphocytes.

**Table 5:** Characteristics of 279 patients and external validation data set.

Model Characteristics		Modeling data(n=279)	Validation data(n=50)	p
Ki-67 expression(%)		30 (16, 45)	33 (20, 50)	>0.1
sTILs (%)		20 (10, 30)	20 (15, 30)	>0.05
Tumor posterior echo (US)	Attenuation	75 (27%)	18 (36%)	>0.05^b^
	Unchanged	201 (72%)	30 (60%)	
	Enhancement	3 (1.1%)	2 (4.0%)	
Molecular subtypes	ER/PR- HER2-	60 (22%)	11 (22%)	>0.1^a^
	ER/PR+ HER2-	102 (37%)	15 (30%)	
	ER/PR- HER2+	48 (17%)	11 (22%)	
	ER/PR+ HER2+	69 (25%)	13 (26%)	

Data format: P50 (P25, P75), (n,%); a: Pearson’s Chi-squared test, b: Fisher’s Exact Test.

### Predictive model of pCR with CTC detection

CTCs were detected pre- and post-NAC in the subgroup of 71 patients (**Table 6**). CTC-positivity decreased from 52.1% (37/71) at baseline to 23.9% (17/71) post-NAC (p < 0.05). Although no initial association between CTCs and pCR was observed (p = 0.173), a significant post-NAC difference emerged between CTC-positive and -negative patients in pCR rates (p = 0.002). Remarkably, patients converting from CTC-positive to CTC-negative after NAC had a significantly increased pCR rate (p = 0.001).

**Table 6:** The relationship between CTCs and pCR rate.

CTCs		N (71)	Non-pCR (n,%)	pCR (n,%)	χ^2^	p
Before NAC	Negative	34	27(79.4%)	7(20.6%)	1.853	0.173
	Positive	37	24(64.9%)	13(35.1%)		
After NAC	Negative	54	34(63.0%)	20(37.0%)	/	0.002^b^
	Positive	17	17(100.0%)	0(0.0%)		
The changes of CTC before and after NAC	Negative/negative	30	23(76.7%)	7(23.3%)	14.412	0.001^b^
	Negative/positive	4	4(100.0%)	0(0.0%)		
	Positive/negative	24	11(45.8%)	13(54.2%)		
	Positive/positive	13	13(100.0%)	0(0.0%)		

CTC, circulating tumor cell; NAC, Neoadjuvant chemotherapy; b, Fisher’s Exact Test.

**Figure 3:**
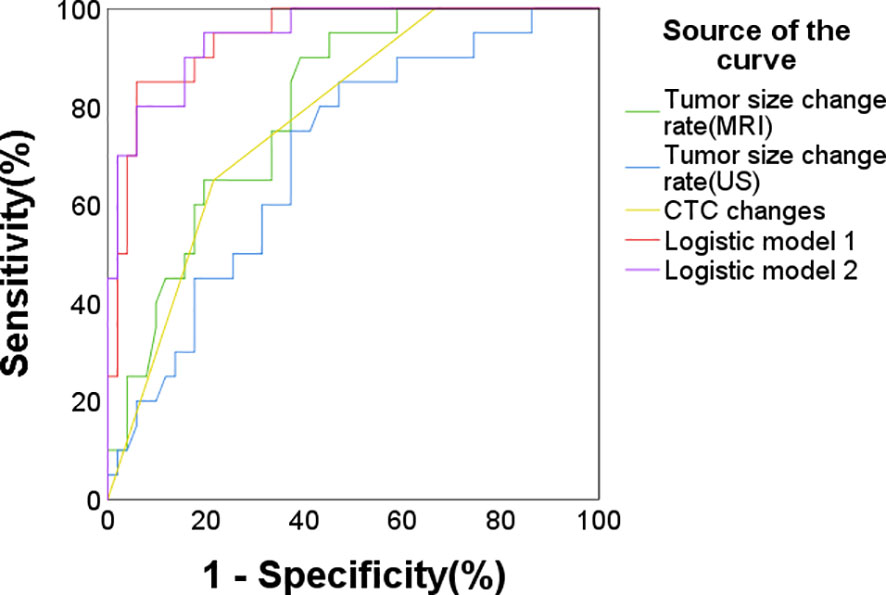
ROC curve for pCR prediction in the CTC subgroup (n = 71). Logistic predictive model 1 (AUC/C-statistics = 0.942), Logistic predictive model 2 (AUC/C-statistics = 0.945) and the components factors: CTC changes (AUC/C-statistics = 0.775), tumor size changes under ultrasound (AUC/C-statistics = 0.7), tumor size changes under MRI (AUC/C-statistics = 0.798), sTIL levels (AUC/C-statistics = 0.838).

In the CTC detection subgroup, the multivariate logistic model considering CTC changes, tumor size changes under US, and sTIL levels achieved a C-statistics/AUC of 0.942 (95% CI: 0.889-0.995) for pCR prediction. Similarly, incorporating changes in tumor size as measured by MRI into the model yielded a comparable predictive performance with a C-statistics/AUC of 0.945 (95% CI: 0.894-0.997) (**Figure 3**, **Table 7**). The C-statistics/AUC values for CTC changes, tumor size changes under US, tumor size changes under MRI, and sTIL levels were 0.775, 0.7,0.798, and 0.838, respectively. The marginal increase in predictive accuracy offered by incorporating MRI-assessed tumor size changes, as evidenced by the comparable C-statistics/AUC values, suggests a limited additional value and cost-effectiveness in utilizing MRI.

**Table 7:** ROC analysis of the Logistic model and 4 factors for prediction of pCR(n=71).

Characteristics	C-statistics/AUC	95%CI	Sensitivity	Specificity	p
CTC changes	0.775	0.665-0.886	0.65	0.784	<0.001
Tumor size changes (US)	0.7	0.571-0.830	0.8	0.569	0.009
Tumor size changes (MRI)	0.798	0.694-0.902	0.9	0.608	<0.001
sTILs	0.838	0.741-0.935	0.5	0.961	<0.001
Logistic model 1 (sTILs + CTC changes + size changes under US)	0.942	0.889-0.995	0.85	0.941	<0.001
Logistic model 2 (sTILs + CTC changes + size changes under MRI)	0.945	0.894-0.997	0.95	0.804	<0.001

CTC, circulating tumor cell; sTILs, stromal tumor infiltrating lymphocytes: Low (sTILs ≤ 10%), Intermediate (10<sTILs<40%), High (sTILs≥40%); US, ultrasound; MRI. magnetic resonance imaging.

### Survival-related data analysis in the cohort

**Figure 4:**
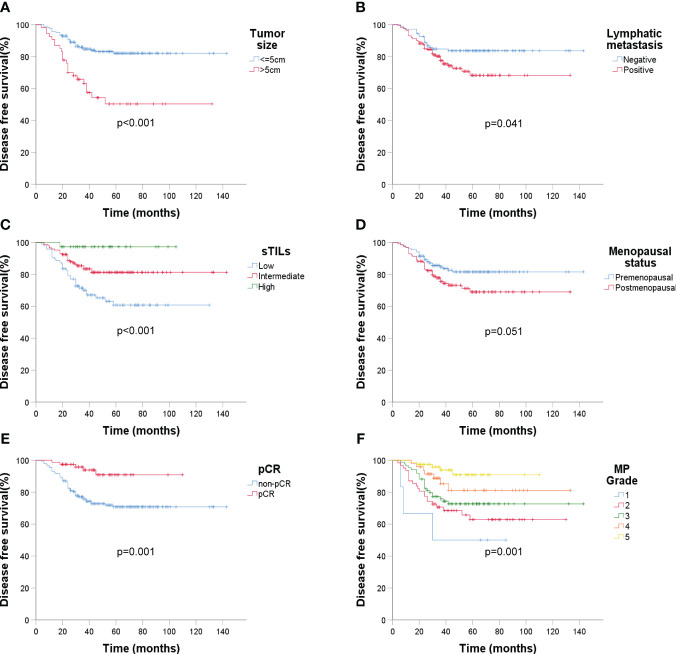
Kaplan-Meier estimates of DFS according to baseline ultrasound, sTIL levels, menopausal status, and Miller-Payne grade after surgery (n = 279). **(A)** DFS rate of tumor size under ultrasound, *p* < 0.001; **(B)** DFS rate of lymphatic metastasis (ultrasound), *p* = 0.041; **(C)** DFS rate of sTIL levels, *p* < 0.001; **(D)** DFS rate of menopausal status, *p* = 0.051; **(E)** DFS rate of pCR, *p* = 0.001; **(F)** DFS rate of MP grade, *p* = 0.001.

**Figure 5:**
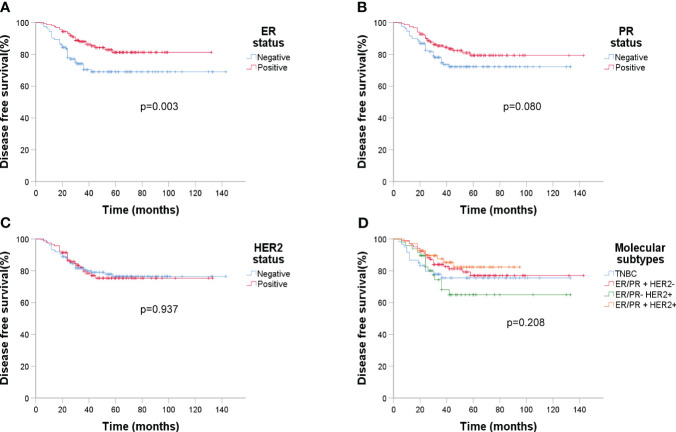
Kaplan-Meier estimates of DFS according to molecular subtypes (n=279). **(A)** DFS rate of ER status, *p* = 0.003; **(B)** DFS rate of PR status, *p* = 0.080; **(C)** DFS rate of HER2 status, p = 0.937; **(D)** DFS rate of four molecular subtypes, *p* = 0.208.

Over an average follow-up of 46.6 months (ranging from 4 to 143 months), a recurrence rate of 20.8% (58/279) was observed by December 2022. Kaplan-Meier curves indicated that larger tumor sizes (>5cm) and presence of lymphatic metastasis were associated with poorer DFS (p < 0.001 and p = 0.041 respectively) (**Figures 4A, B**). Higher sTIL levels were linked with a reduced recurrence risk (p < 0.001) (**Figure 4C**). Interestingly, premenopausal patients had a slightly longer DFS (p = 0.051) (**Figure 4D**). A clear association between pCR and better DFS emerged (p = 0.001) (**Figures 4E, F**), but molecular subtypes didn’t significantly affect recurrence (**Figure 5**).

**Figure 6:**
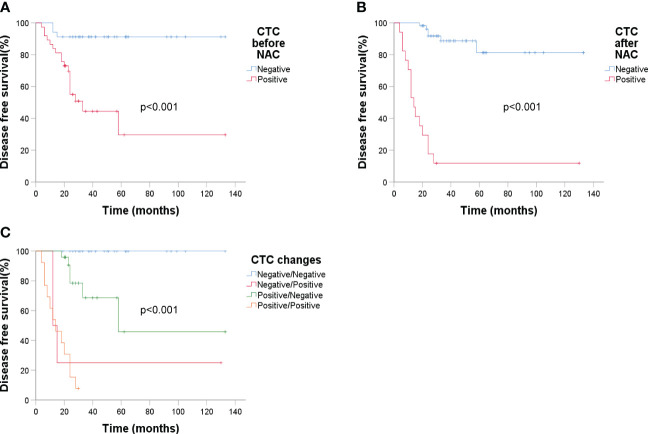
Kaplan-Meier estimates of DFS according to circulating tumor cells (n = 71). **(A)** DFS rate of baseline CTC, *p* < 0.001; **(B)** DFS rate of CTC after NAC, *p* < 0.001; **(C)** DFS rate of CTC changes before and after NAC, *p* < 0.001.

**Figure 7:**
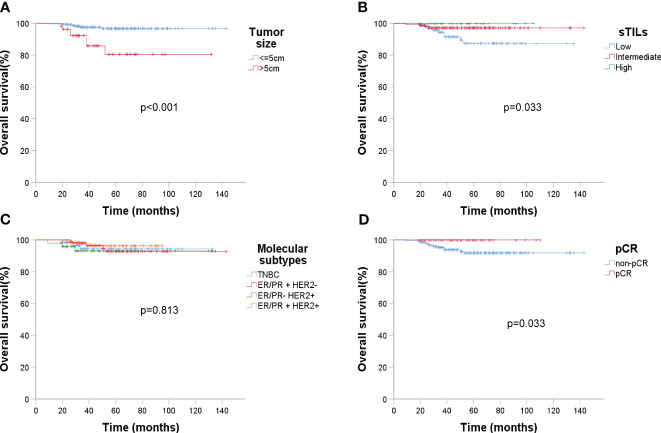
Kaplan-Meier estimates of overall survival (OS) (n = 279). **(A)** OS rate of baseline tumor size under ultrasound, *p* < 0.001; **(B)** OS rate of sTIL levels, *p* = 0.033; **(C)** OS rate of molecular subtypes, *p* = 0.813; **(D)** OS rate of pCR, *p* = 0.033.

ER status had a significant impact on recurrence (p = 0.003). Analyzing DFS against CTC data (**Figure 6**) revealed that post-NAC CTC positivity correlated significantly with poorer DFS (p < 0.001). Finally, Kaplan-Meier plots for overall survival exhibited similar patterns regarding baseline tumor size, sTIL levels, and pCR (**Figure 7**).

## Discussion

The prediction and understanding of the pCR and subsequent prognosis after NAC in breast cancer remains a significant challenge in clinical practice. The pursuit of an optimal predictive model for both pCR and prognosis is paramount, as it holds the potential to guide therapeutic decisions and enhance personalized patient care.

In this study, we integrated an array of clinical, pathological, and biomarker features to devise a comprehensive predictive model for pCR following NAC in breast cancer patients. Our integrated model, combining sTIL levels, Ki-67 expression, molecular subtypes, and ultrasound echo characteristics, demonstrated significant predictive power with an impressive C-statistics/AUC of 0.874. Its robust performance was further affirmed in the external validation set, achieving a C-statistics/AUC of 0.836, thereby underscoring the model’s efficacy. Among the predictors, sTIL levels stood out as the most influential factor. While previous research has indeed underscored the correlation of sTIL levels with prognosis in tumor tissues (31–33), their relationship and prognostic value across different molecular breast cancer subtypes remain nuanced (34, 35). For instance, while elevated sTIL levels have shown favorable prognosis in TNBC (36, 37), their significance in other molecular subtypes, especially ER/PR+ HER2-, is less consistent (34, 38). Our findings further highlight this molecular subtype-specific relationship, emphasizing the nuanced role of sTILs in predicting outcomes. The relationship between sTIL levels and prognosis across different molecular subtypes in breast cancer, and its value in prediction and evaluating treatment warrant further clarification.

In addition to incorporating pre- and post-NAC dynamics of CTCs, our model highlighted the significant predictive capacity of combining CTC changes with US-assessed tumor size alterations and sTIL levels, achieving a C-statistics/AUC of 0.942. The model incorporating MRI for tumor size changes showed a similar predictive performance. The C-statistics/AUC of the model with US and the model with MRI were closely comparable, being 0.942 and 0.945 respectively. Considering the complexity and higher cost associated with MRI, ultrasound emerges as the more practical and cost-effective evaluation method in this predictive model. The observed strong correlation between post-NAC CTC positivity with both non-pCR and decreased DFS highlights the utility of CTCs as a prognostic marker. Often referred to as ‘liquid biopsies’, CTCs are rapidly gaining recognition as vital components in cancer management, known for their roles in early detection, prognostic evaluation, and monitoring for recurrence (39–41). Our study reinforces their significance in breast cancer, underscoring their potential as key predictors for both pCR and overall prognosis.

In addition to the above features, our data accentuated traditional clinical features, such as large tumor sizes (> 5 cm) and lymph node metastasis, remain crucial in prognosis. Furthermore, low sTIL levels and negative ER expression were all linked with poor DFS, indicating their potential as prognostic markers, and providing a holistic view of the complex prognostic landscape of breast cancer.

One intriguing observation was the differential pCR rates across molecular subtypes. While the ER-PR- HER2+ subtype exhibited a higher rate of pCR, the ER/PR+ HER2- group showed a lower rate. However, when assessing DFS post-NAC, no significant differences were observed between these molecular subtypes. This raises pertinent questions about the interplay between short-term treatment responses and long-term survival outcomes, suggesting that achieving pCR does not always equate to improved long-term prognosis.

Our study contributes to the broader understanding of pCR and prognosis in breast cancer; however, it is not without its limitations. The sample size, especially within the CTC subgroup, may limit the generalizability of our findings, and the retrospective design of our analysis could potentially influence the results. These constraints underscore the necessity for larger, prospective studies to confirm and expand upon our findings. Looking ahead, we envision a comprehensive future trial that would not only validate our current results but also enhance the predictive model for broader clinical utility.

## Conclusions

Our study has developed a sophisticated model adeptly predicting pCR post-NAC in breast cancer, integrating sTILs, Ki-67 expression, molecular subtypes, and ultrasound features with a C-statistics/AUC of 0.874. Particularly in the CTC-detected subgroup, the model combining CTC changes, tumor size changes, and sTILs achieved an impressive C-statistics/AUC of 0.945. Key determinants like tumor > 5 cm, presence of lymph node metastasis, reduced sTILs, and negative ER expression were identified as pivotal to diminished DFS. Essentially, our findings offer an enriched pCR prediction model, highlight salient factors affecting prognosis, and underscore the potential for individualized breast cancer treatments.

## Data availability statement

The original contributions presented in the study are included in the article/**Supplementary Material**. Further inquiries can be directed to the corresponding author.

## Ethics statement

The studies involving humans were approved by the ethics committees of all institutions involved. The studies were conducted in accordance with the local legislation and institutional requirements. The human samples used in this study were acquired from a by- product of routine care or industry. Written informed consent was obtained for Circulating Tumor Cell (CTC) detection from the participants. For other aspects of the study not related to CTC detection, written informed consent was not required from participants or their legal guardians/next of kin, in line with national and institutional regulations.

## Author contributions

SF: Data curation, Formal analysis, Funding acquisition, Writing – original draft, Writing – review & editing. WX: Conceptualization, Data curation, Funding acquisition, Writing – original draft, Writing – review & editing. HZ: Formal analysis, Writing – review & editing. CN: Funding acquisition, Validation, Writing – review & editing. JW: Writing – original draft. QM: Investigation, Writing – review & editing. MJ: Funding acquisition, Writing – review & editing. DG: Data curation, Writing – review & editing. HY: Project administration, Supervision, Writing – review & editing. WC: Conceptualization, Writing – original draft.

## Supplementary material

Supplementary Figure 1 ROC curve of the prediction for pCR with sTIL levels, Ki-67expression and tumor posterior echo in 4 molecular subtypes. TNBC(ER- PR- HER2-): AUC/C-statistics = 0.870; ER/PR+ HER2-: AUC/C-statistics = 0.810; ER- PR- HER2+: AUC/C-statistics = 0.878; ER/PR+ HER2+: AUC/C-statistics = 0.780.
