# Supplementary material for: A real-world clinicopathological model for predicting pathological complete response to neoadjuvant chemotherapy in breast cancer
Source: Front Oncol. 2024 Feb 14;14:1323226. doi: 10.3389/fonc.2024.1323226 (PMC10899694; doi:10.3389/fonc.2024.1323226)
Supplement: Supplementary Figure 1 — ROC curve of the prediction for pCR with sTIL levels, Ki-67expression and tumor posterior echo in 4 molecular subtypes. TNBC(ER- PR- HER2-): AUC/C-statistics = 0.870; ER/PR+ HER2-: AUC/C-statistics = 0.810; ER- PR- HER2+: AUC/C-statistics = 0.878; ER/PR+ HER2+: AUC/C-statistics = 0.780. [file Image_1.pdf]

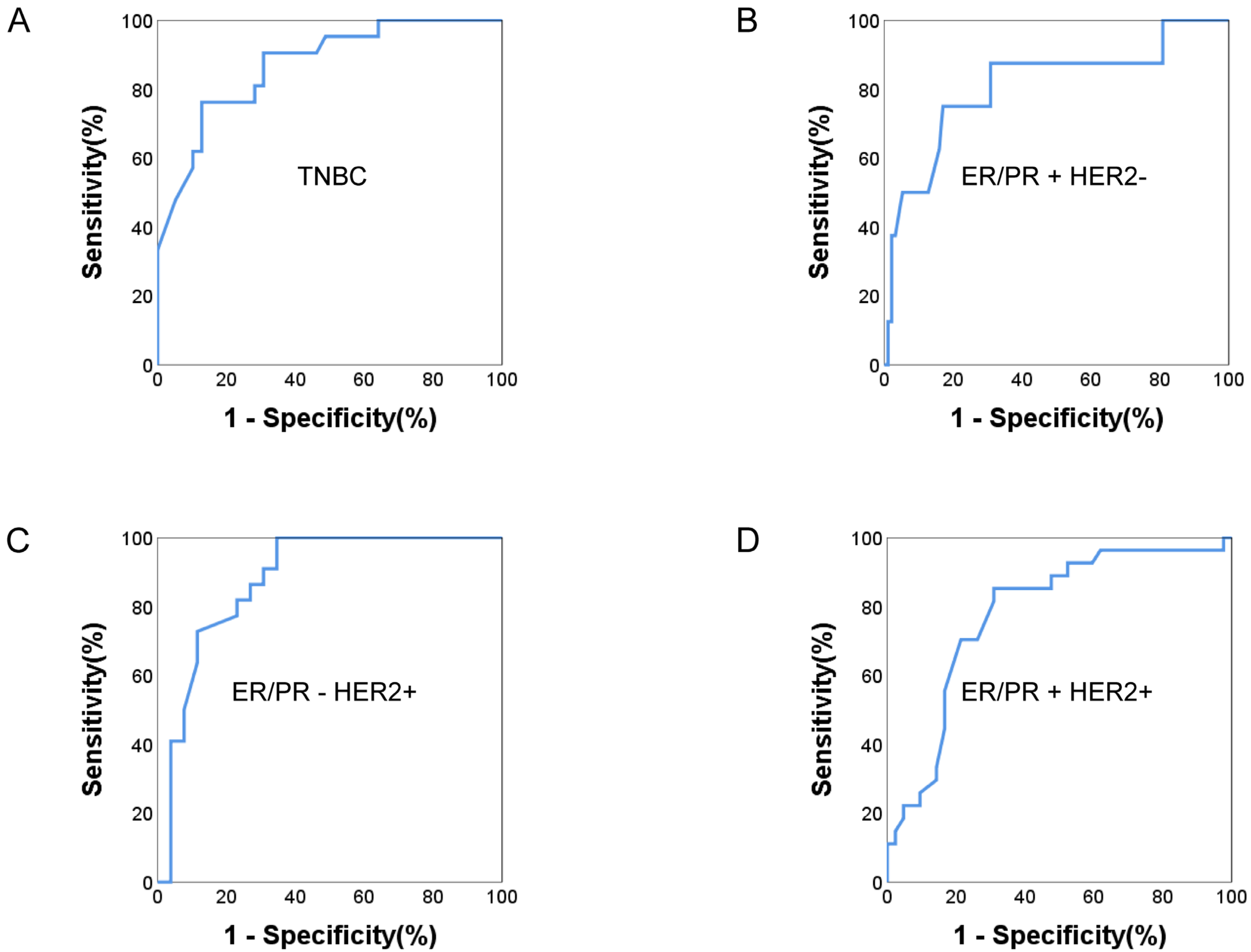
A
TNBC
B
ER/PR + HER2-
C
ER/PR - HER2+
D
ER/PR + HER2+
Sensitivity(%)
1 - Specificity(%)
0
20
40
60
80
100

- **Figure S1： ROC curve of the prediction for pCR with TILs, Ki-67expression and posterior echo of US in four molecular subtypes; TNBC: AUC = 0.870; ER/PR + HER2- : AUC = 0.810; ER-PR- HER2+: AUC = 0.878; ER/PR + HER2+: AUC = 0.780.**
